# Diversity and community assembly mechanisms of soil methanotrophs in typical ecotypes of the Mitika alpine wetland in northern Xizang

**DOI:** 10.3389/fmicb.2025.1731213

**Published:** 2025-12-08

**Authors:** Pengxi Cao, Yuyan Wang, Zhe Wang, Liang Cao, Hairong Li, Hongmei Ma, Qiong La

**Affiliations:** 1 Key Laboratory of Biodiversity and Environment on the Qinghai-Tibetan Plateau, Ministry of Education, School of Ecology and Environment, Xizang University, Lhasa, China; 2 Nagqu Mitika, Alpine Wetland Ecosystem, Observation and Research Station of Xizang Autonomous Region, Xizang University, Nagqu, China; 3 Collaborative Innovation Center for Ecological Civilization of the Qinghai-Xizang Plateau, Xizang University, Lhasa, China

**Keywords:** methanotrophs, seasonal dynamics, alpine wetland, community diversity, environmental factors

## Abstract

The Mitika alpine wetland is a globally important wetland on the Qinghai-Xizang Plateau, which serves as a vital carbon reservoir on Earth’s surface. However, the seasonal variation characteristics of its soil methanotrophs communities remain poorly understood. To deepen our understanding of the role of this biome in geochemical cycles, we selected three typical ecosystems from the Mitika wetland in northern Xizang: alpine grassland, swamp meadow, and fen. Surface soil samples were collected across spring, summer, autumn, and winter seasons. Using high-throughput sequencing, we analyzed methanotrophs diversity, community structure, and responses to environmental factors. The dominant phyla were *α-Proteobacteria* (Type II aerobic methanotrophs), candidate_division_NC10, and unclassified bacteria. At the genus level, *Methylocystis*, *Methylococcus*, and *Methylocapsa* were the primary taxa. Neutral model analysis indicated that random processes dominate community assembly, with winter communities better fitting the neutral model and exhibiting lower diffusion constraints. Among environmental factors, pH, Total Nitrogen (TN), Electrical Conductivity (EC), Salt (Salt), and Water Content (WC) showed significant correlations with certain methanotrophs groups. Structural equation modeling further revealed that fundamental soil physicochemical factors exert a significant positive influence on alpha diversity. Our findings reveal the seasonal dynamics and ecosystem differences of methanotrophs communities in the Mitika alpine wetland, thus contributing to a more thorough understanding of carbon cycling functions in alpine wetlands.

## 1 Research background

Wetlands are among the most critical terrestrial habitats on Earth. Spanning both terrestrial and aquatic environments, they possess unique hydrological, soil, vegetation, and biological characteristics. As they are among the most biodiverse ecological landscapes and critical habitats for wildlife, wetlands play vital roles in regulating runoff, mitigating floods, and improving water quality, while also providing essential habitats for numerous plants and animals. Thus, wetlands are crucial for maintaining ecosystem balance and safeguarding biodiversity (Junk et al., 2013; Guo et al., 2017; Mahdavi et al., 2018). The breadth of wetland distribution in China and associated biological resources are globally important (Zhao, 1982; Harrison et al., 1995). The Qinghai-Xizang Plateau accounts for approximately one-third of the total wetland area in China (Zhu et al., 2021), with its unique alpine wetlands playing an indispensable role in regional ecological security (Wei et al., 2020; Zhang et al., 2023). Primarily distributed in water-rich areas such as riverbanks and lakeshores, these wetlands endure prolonged ice and snow cover, forming a specialized wetland type adapted to alpine climatic conditions (Chen et al., 2002). The Qinghai-Xizang Plateau hosts the most extensive distribution of alpine wetlands in the region, predominantly comprising fen and alpine meadows. Most of the wetlands are located in Xizang and Qinghai, where they provide critical environmental services, particularly water supply replenishment and regional climate regulation. These wetland areas play an irreplaceable role in maintaining regional ecological balance and preserving biodiversity, while also delivering significant ecological and economic benefits (Feng and Cheng, 2014).

Key aspects of global change include atmospheric composition changes, global climate change, sea level rise, biodiversity shifts, biological invasions, and alterations in land use and land cover (Peng et al., 2020). Since the 19th century, the long-term loss rate of global natural wetlands has ranged between 64 and 71% (Davidson, 2014). Enhanced evaporation due to climate warming caused continuous wetland degradation on the Qinghai-Xizang Plateau prior to 2000, with wetland areas decreasing at an annual rate of 0.15%, resulting in a total reduction of 2,804.63 km^2^ (Chen et al., 2021). In this context, research on the response of the alpine wetlands in China to global climate change is of critical importance. Globally, all economies depend on the goods and services provided by the natural environment. As a natural resource, soil performs numerous critical environmental, social, and economic functions (Blum, 2005). Soil is central to the biotransformation of organic carbon and continuously contributes to the release of CO₂ and other trace gasses into the atmosphere. Furthermore, soil is a vital foundation for maintaining biodiversity, harboring more species than all other terrestrial biota combined (Li et al., 2024; Wang, 2024). Therefore, comprehensive analysis of soil physicochemical properties is warranted in order to establish scientific guidance for the rational utilization and conservation of land resources.

Soil microorganisms are central to maintaining soil functions and services, representing the largest terrestrial carbon reservoir and storing over 90% of global soil carbon (Grosskopf et al., 1998; Chen et al., 2022). The impacts of global change on alpine wetlands may primarily affect soil microbial functions, thereby further influencing ecosystem carbon and nitrogen cycles. For example, rising temperatures could lead to a global decline in soil microbial carbon. Current research on alpine wetlands in China primarily focuses on global change (Zhou, 2022), carbon and nitrogen cycles (Wang et al., 2023), and functional microbial communities (Guo et al., 2022). The Earth system carbon cycle primarily refers to the circulation of carbon among the three major reservoirs: atmosphere, oceans, and land. Globally, these reservoirs can be divided into four relatively independent compartments: biota, rock/soil, terrestrial and marine water bodies, and atmosphere (Miller et al., 2019). On Earth, carbon exists in the atmosphere as carbon dioxide (CO₂) and in the oceans as dissolved forms such as bicarbonate (HCO₃^−^) and carbonate (CO₃^2−^), and in the biosphere, where it is primarily stored as organic matter. Soil carbon stocks are estimated to reach 1,270 Gt, which is two to three times higher than atmospheric CO₂ concentrations (Chen et al., 1998). Consequently, even minor changes in the soil carbon pool can substantially impact soil health and land productivity and may further influence global climate change (Lal, 2004).

As the core of terrestrial ecosystems, soil serves as a crucial link connecting the atmosphere, hydrosphere, biosphere, and lithosphere. Therefore, understanding soil carbon cycling processes is fundamental to studying carbon cycling in terrestrial ecosystems (Gao, 2006) investigated changes in soil organic carbon (SOC) stocks in the Ruoergai Plateau wetlands. Using the Environmental Policy Integrated Climate model; the authors quantitatively analyzed SOC response changes from 1980 to 2010 in two counties of the Ruoergai Plateau wetlands, where gradual drainage and increased grazing intensity had occurred. Kang et al. (2016) argued that quantifying phenological changes and variability in gross primary productivity of alpine wetlands on the Xizang Plateau under climate change is crucial for assessing regional and global carbon balance dynamics.

Methanotrophs utilize methane as their sole carbon source and energy for catabolic and anabolic metabolism (Strong et al., 2015). Functioning as biological methane filters within wetland ecosystems, they have garnered significant scholarly attention. These microorganisms play crucial ecological roles in nature, particularly within the biogeochemical cycle of methane (Deng et al., 2015). Methanotrophs play a vital role in wetland ecosystems. Methane emissions from wetlands result from the combined processes of methane production, transport, and oxidation, with emission levels closely linked to the activity of methanogenic and methanotrophs. The diversity of methanotrophs is reflected in their extensive phylogenetic distribution, spanning from bacteria to archaea across multiple phyla-level taxonomic units. They also exhibit metabolic versatility, utilizing oxygen, sulfate, nitrate, nitrite, metals, and other compounds as electron acceptors (Guerrero-Cruz et al., 2013). By regulating these microbial communities in wetlands, methane metabolism can be effectively controlled, reducing methane emission fluxes and contributing to mitigating global climate change. Studies reveal significant differences in community structure among various peatland types, with methanotroph metabolic activity also varying across latitudes. pH emerges as the strongest predictor of peatland microbial community structure, with wetlands under different pH conditions selecting distinct methanotrophic bacterial communities (Seward et al., 2020). In a study by Zhang field control experiments were conducted. Research on how precipitation pattern changes affect carbon emissions in alpine paludified meadow ecosystems remains scarce, and understanding of the underlying mechanisms is unclear (Zhang, 2018). This severely limits our ability to comprehend and predict the ecological consequences of climate change and develop adaptive strategies accordingly. The study revealed that the high-altitude marshy meadow in Ruoergai serves as a methane sink. Severe alterations in precipitation patterns led to reduced soluble organic carbon concentrations and altered microbial communities, which in turn weakened soil methane absorption capacity and caused an estimated 54.3% decline in soil methane uptake (Zhang et al., 2022). In a study by Zhang et al. (2022), high-throughput sequencing of the *mcrA* gene analyzed the community structure and diversity of methanogenic bacteria in alpine wetland soils on the Xizang Plateau. The authors employed open-top chambers to simulate warming experiments, investigating the impact of temperature increase on the community structure and diversity of methanotrophs in lakeside wetlands (Sitch et al., 2003; Li et al., 2020). In a study by Zhang Yanfen, the methanogenic functional microorganisms in soils of three dominant plant species in Ruoergai alpine wetlands were investigated. The results indicated high abundance of methanogenic microorganisms in these wetlands and clarified their primary community structure composition (Zhang, 2018). Research by Mo et al. revealed, through a 90-day stable isotope tracing study combined with high-throughput sequencing technology, that aerobic methanotrophs Methylobacter play a central role in the suboxidative environment of soils in alpine fens on the Tibetan Plateau. This bacterial group not only serves as the key functional community driving methane oxidation under hypoxic conditions but also likely acts as the primary executor of coupled nitrogen fixation processes. This discovery challenges the conventional understanding that aerobic processes strictly depend on high oxygen concentrations, revealing the robust adaptive strategies of aerobic bacteria like Methylobacter in dynamically hypoxic environments and their critical ecological role in coupling wetland carbon and nitrogen cycles (Mo et al., 2020).

However, a systematic understanding of the seasonal dynamics of soil methanotrophs communities and their variations across different ecosystems in the Mitika alpine wetland remains lacking. Therefore, this study aims to address the following core scientific question: How do the diversity, community structure, and assembly processes of soil methanotrophs in the Mitika alpine wetland respond to seasonal dynamics and shifts across typical ecosystem types? Based on this, we propose the following hypotheses: (1) The methanotrophs community structure and diversity significantly differ across ecosystem types and seasons; (2) Stochastic processes dominate the assembly of methanotrophs communities in this region, and the strength of these processes varies with season and ecosystem type; (3) Fundamental soil physicochemical properties are key environmental factors governing community structure and diversity. By addressing these questions, this study aims to provide a microbiological basis for a deeper understanding of the role of alpine wetlands in the global carbon cycle.

## 2 Methods

### 2.1 Overview of the study area

**Figure 1:**
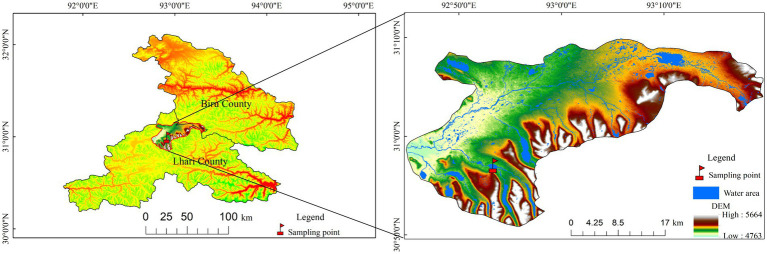
Overview of the study area.

The Mitika Wetland National Nature Reserve is located in the township of Mitika, Jiali County, Nagqu Prefecture, northern Xizang, China (Figure 1). The geographic coordinates of the study area range from 30°51′04″ to 31°09′44″N and 92°45′55″ to 93°19′25″E. The reserve covers a total area of 88,052.37 ha, with an average elevation is 4,917.6 m. The climate is classified as a subarctic semi-humid plateau climate, characterized by diverse weather patterns, indistinct seasons, and pronounced cold and warm periods. The annual average temperature ranges from −1.7 to 0.7 °C, with the coldest months being December and January, and the warmest temperatures typically occurring in July. Annual precipitation averages around 700 mm, concentrated primarily from June to September. Annual evaporation is approximately 1,400 mm, and the annual average sunshine duration is 2211.8 h. Primary soil types include alpine desert soils, alpine meadow soils, marsh soils, as well as alluvial and coarse-textured soils. Major meteorological hazards include heavy winter and spring snowfall, wind disasters, blizzards, hailstorms, frost, sudden temperature drops, and thunderstorms. Plants found in the reserve that are endemic to Xizang include *Corydalis chrysosphaera*, *Rheum rhomboideum*, *Potentilla turfosa* var. *gracilescens*, *Androsace graminifolia*, and *Phlomoides younghushandii* (Li et al., 2017).

### 2.2 Sample collection

Soil samples were collected during different seasons in May, August, and December 2023, and March 2024 at the Wupiancuo study area in the Mitika Wetland, Xizang. Three typical ecosystem types were selected: alpine grassland, swamp meadow, and fen (Table 1). For each ecosystem type, three replicate plots (1 × 1 m each) were established randomly, resulting in a total of nine plots. Soil samples were collected from the 0–10 cm depth after removing surface vegetation and debris. The five-point sampling method was employed within each plot, and subsamples from the five points were thoroughly mixed to form one composite sample per plot. Sampling in summer and autumn was conducted using an 8 cm diameter soil sampler. In contrast, due to frozen soil conditions, the Christie SD-1 soil drilling machine was utilized in spring and winter. The weight of each composite soil sample was approximately 100 g. In total, 36 soil samples were collected (4 seasons × 3 ecosystems × 3 replicates = 36 samples). Soil samples were placed in sterile bags, thoroughly mixed to represent the plot, and labeled with identification information such as sampling time, location, and sample number. A total of 36 soil samples were collected. Samples were placed in a vehicle-mounted refrigerator at −4 °C for transport back to the laboratory for processing. The soil samples were divided into two portions. One portion was sieved through a 2 mm diameter mesh to remove roots, sand, gravel, and other impurities as thoroughly as possible. This portion was dried in a 95 °C oven for 24 h and subjected to standard physical–chemical analysis. The other portion was frozen at −80 °C for subsequent extraction of total soil DNA.

**Table 1:** Environmental parameters in Mitika across different seasons.

Seasons	Average air pressure (hpa)	Soil temperature (°C)	Air temperature (°C)	Concentration (ppm)	Light intensity (Lux)	Average wind speed (m/s)
Spring	559.57	−2.36	−1.48	438.63	40746.14	4.55
Summer	563.82	4.96	7.44	439.11	48548.75	3.62
Autumn	565.26	8.99	14.24	415.14	43568.32	3.24
Winter	561.27	−3.18	−10.67	430.90	24498.13	3.84

### 2.3 Soil physicochemical analysis

All soil physicochemical properties were determined using 20 g fresh soil samples. The specific indicators measured and their corresponding standard analytical methods are as follows: pH: Measured potentiometrically in a 1:1 (w/w) soil-water suspension. Electrical Conductivity (EC): Measured conductometrically in a 1:3 (w/w) soil-water suspension. Water Content (WC): Determined by the gravimetric method after drying at 105 °C to a constant weight. Salinity (Salt): Determined directly from the soil extract using a salinity meter (PCSTestr 35). Organic Matter Soil Organic Matter (SOM): Determined by the potassium dichromate oxidation method with external heating. Total Carbon (TC): Determined by the dry combustion method using an elemental analyzer. Total Nitrogen (TN): Determined by the Kjeldahl digestion method. Alkali-hydrolyzable Nitrogen Alkali-hydrolyzed Nitrogen (HN): Determined by the alkali diffusion method. Ammonium Nitrogen (AN): Extracted with potassium chloride and determined by the indophenol blue colorimetric method. Nitrate Nitrogen (NN): Extracted with calcium chloride and determined by dual-wavelength ultraviolet spectrophotometry. Available Phosphorus (AP): Extracted with sodium bicarbonate and determined by the molybdenum-antimony anti-colorimetric method. Available Potassium (AK): Extracted with ammonium acetate and determined by flame photometry.

### 2.4 DNA sequencing

This study employed 16S rRNA gene sequencing technology to detect and analyze methanotroph communities. DNA Extraction: Place 0.25–0.5 g of soil sample into a 2-mL centrifuge tube. Add 500 μL Buffer SA, 100 μL Buffer SC, and 0.25 g grinding beads. Vortex for 15 min until thoroughly homogenized, or use the TGrinder H24 Tissue Homogenizer (OSE-TH-01) at 6 m/s for 30 s, followed by 30 s rest, repeated for 2 cycles. Centrifuge at 12,000 rpm/min. Transfer the supernatant (approximately 500 μL) to a new 2 mL centrifuge tube. Complete nucleic acid extraction using the TGuide S96 Magnetic Bead-Based Soil Genomic DNA Extraction Kit. Amplify the pmoA gene of methanotrophs using primers pmoAF325 (5’-TGGGGYTGGACCTAYTTCC-3′) and pmoaAR643 (5’-CCGGCRCRACGTCCTTACC-3′) (Kip et al., 2010). The total PCR reaction volume was 20 μL, comprising: 5–50 ng DNA template, 0.3 μL forward primer (10 μM), 0.3 μL reverse primer (10 μM), 5 μL KOD FX Neo buffer, 2 μL dNTPs (2 mM each), 0.2 μL KOD FX Neo enzyme, and ddH₂O to a final volume of 20 μL. The PCR amplification program was as follows: 5 min pre-denaturation at 95 °C, followed by 20 cycles each consisting of 30 s denaturation at 95 °C, 30 s annealing at 50 °C, and 40 s extension at 72 °C, concluding with a 7 min extension at 72 °C. Amplified products were purified using the Omega DNA Purification Kit (Omega Inc., Norcross, GA, USA) and quantified with the Qsep-400 system (BiOptic, Inc., New Taipei City, Taiwan, China). The final amplicon library underwent paired-end sequencing (2 × 250 bp) on the Illumina Novaseq 6,000 platform, performed by Beijing Biomarker Technologies Co., Ltd. (Beijing, China).

### 2.5 Data analysis

Sequenced paired-end reads were assembled and filtered using Flash and Trimmomatic software. Cluster analysis was performed on the resulting data using Usearch software. To determine the feasibility of sample grouping, ANOSIM similarity and Adonis permutation multivariate analysis of variance were applied to the samples. Furthermore, several advanced analytical approaches were implemented to deepen our understanding of microbial community assembly and environmental interactions. The Neutral Community Model (NCM) was fitted to the soil microbial community data using the microbiome and remotes packages in R (v4.4.1) to quantify the relative importance of stochastic processes in community assembly. Structural Equation Modeling (SEM) was constructed using R (v4.4.1) to elucidate the complex relationships between environmental factors, microbial taxa, and ecosystem functions, with significant physicochemical factors selected through Spearman correlation analysis. For environmental factor correlation analysis, we employed Spearman correlation analysis combined with Mantel tests to identify significant associations between microbial community composition and environmental variables, with results visualized using the pheatmap package to generate correlation heatmaps. Analysis of Sobs, Shannon, Simpson, Ace, Chao, and Coverage indices was performed, with Welch’s *t*-test used to assess intergroup differences in alpha diversity. Non-metric multidimensional scaling (NMDS) was employed to analyze microbial community similarity. Partial data from this study were also analyzed using the BioMetic Cloud platform. Supporting data for this study were uploaded to the National Microbial Data Center (NMDC) under accession number NMDC10020266, accessible at https://nmdc.cn/resource/genomics/project/detail/NMDC10020266.

## 3 Results

### 3.1 Analysis of alpha diversity of methanotrophs across seasons

**Figure 2:**
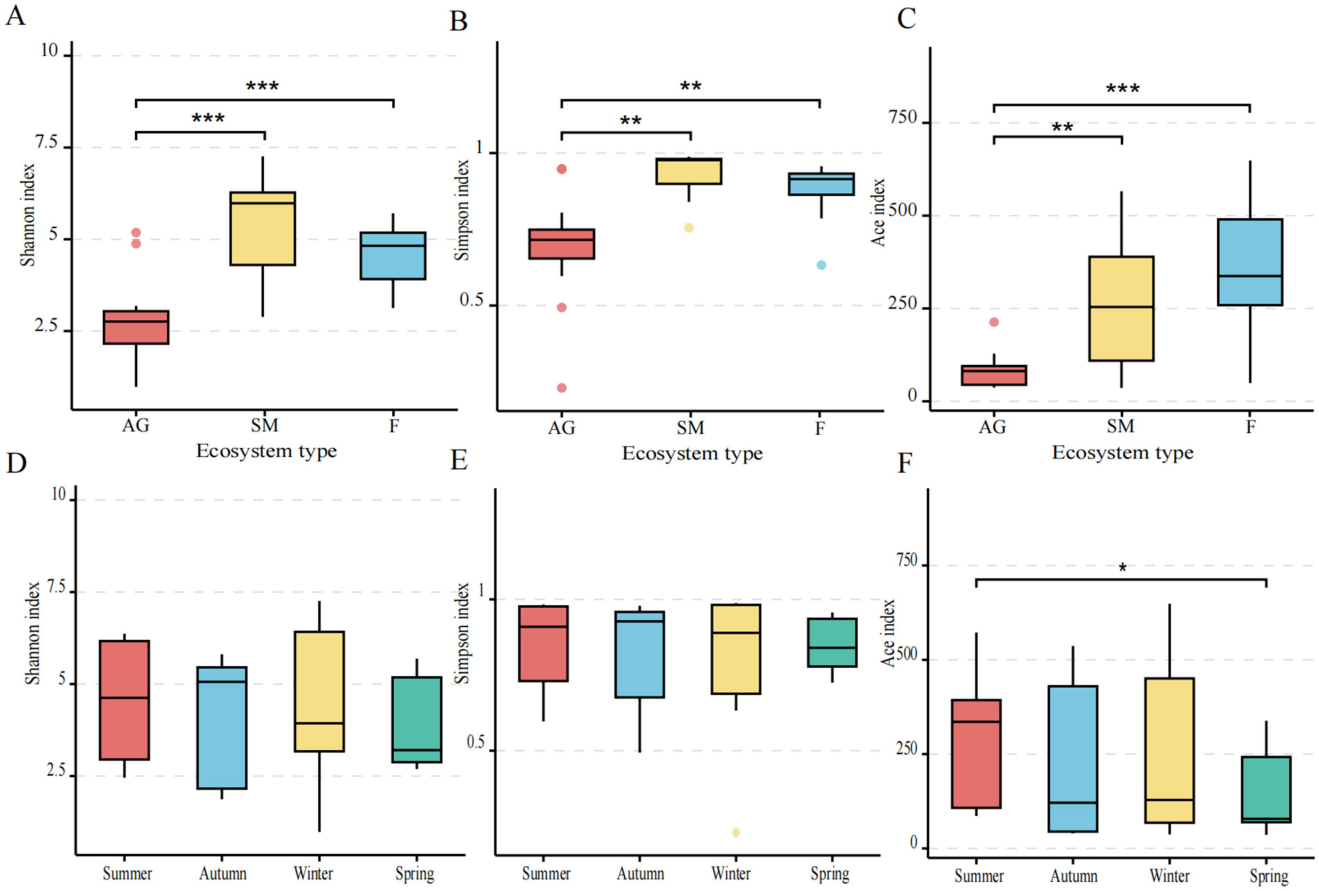
Alpha diversity index of methanotrophs in alpine wetland ecosystems of Mitika in different seasons. **(A–C)** Diversity indices of methanotroph communities in different ecosystems; **(D,F)** diversity indices of methanotroph communities in different seasons (*0.01 < *p* ≤ 0.05, **0.001 < *p* ≤ 0.01).

Alpha diversity of methanotrophs across seasons was assessed using Shannon’s and Simpson’s indices for species diversity and Ace’s index for species richness. Significant differences in Shannon, Simpson, and Ace indices were observed between alpine grassland and fen, as well as between alpine grassland and swamp meadow (Figures 2A–C). Among ecosystems, swamp meadow exhibited the highest diversity, followed by fen, with alpine grassland showing the lowest diversity. Across seasons, the Ace index showed significant differences between spring and summer (*p* < 0.05) but not between other seasons. Across seasons, The Shannon and Simpson indices exhibited no significant seasonal variation (Figures 2D,E). The Ace index showed significant differences between spring and summer (*p* < 0.05) but not between other seasons (Figure 2F).

### 3.2 Beta diversity analysis of methanotrophs across seasons

**Figure 3:**
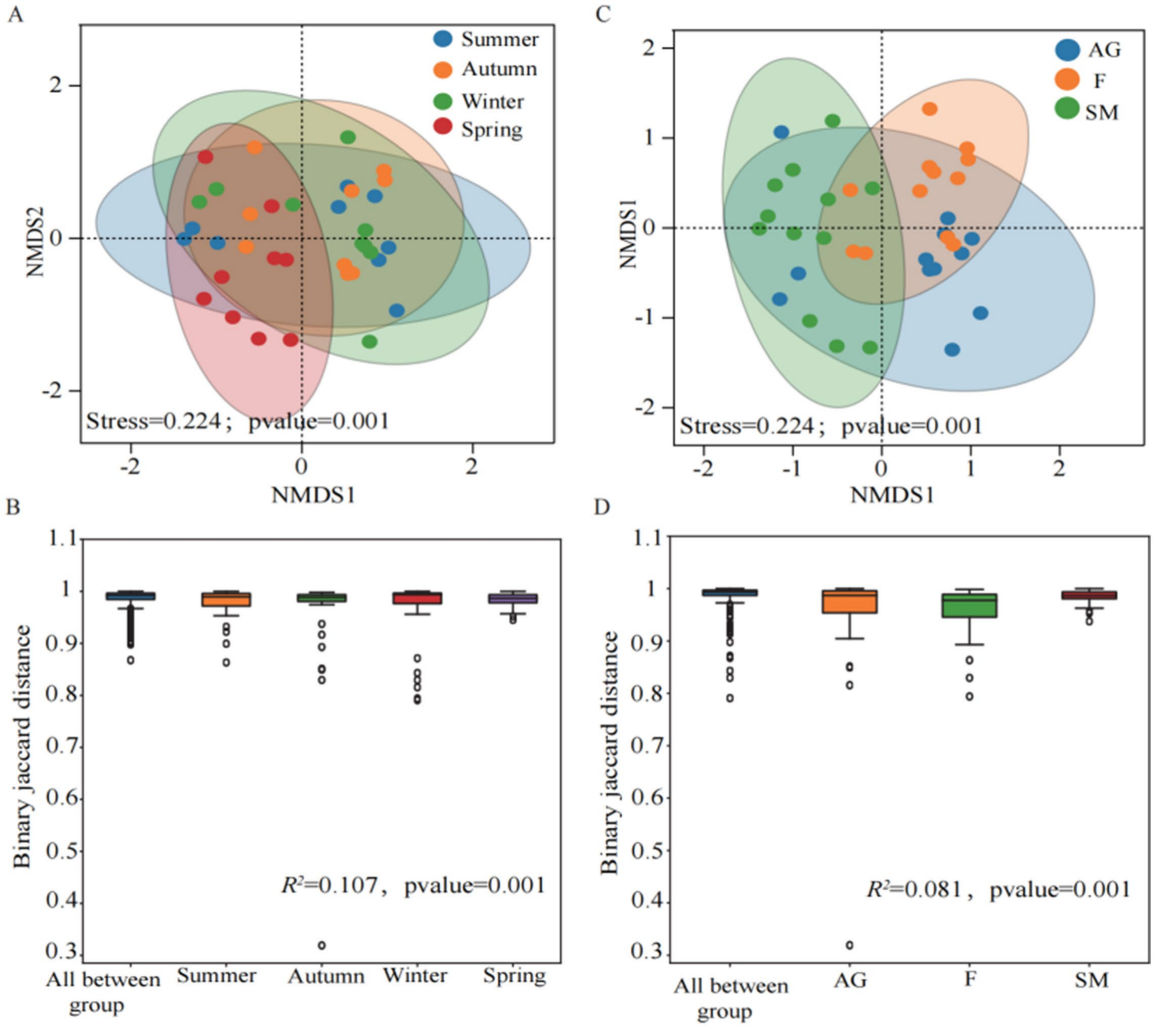
Beta diversity analysis of soil methanotrophs in different seasons **(A,B)** and different ecosystems **(C,D)** in the Mitika alpine wetland.

Beta diversity across seasons was analyzed using PERMANOVA to evaluate grouping variable contributions, with permutation tests for statistical significance validation. NMDS was applied to examine differences in Beta diversity among groups. The results indicated a good model fit. Under the groupings of different seasons and ecosystems, samples exhibited some overlap but also distinct separation, indicating that different seasons and ecosystems exerted certain influences on methanotrophs communities (Figures 3A,B). The binary Jaccard distances between different seasons and ecosystems showed significant differences (*p* = 0.001), further demonstrating that both groupings significantly affected the distribution of methanotrophs communities (Figures 3C,D). That methanotroph community compositions were not significantly different between summer and autumn (*p* > 0.05). In contrast, significant compositional shifts were observed between winter and spring (*p* = 0.001) (Figure 3C). Similarly, community compositions between alpine grassland and swamp meadow were not statistically distinct (*p* > 0.05). However, the fen harbored a significantly different community compared to both the alpine grassland and swamp meadow (*p* = 0.001) (Figure 3D).

### 3.3 Seasonal variation in methanotrophs community structure

#### 3.3.1 Methanotrophs community composition at the phylum level

**Figure 4:**
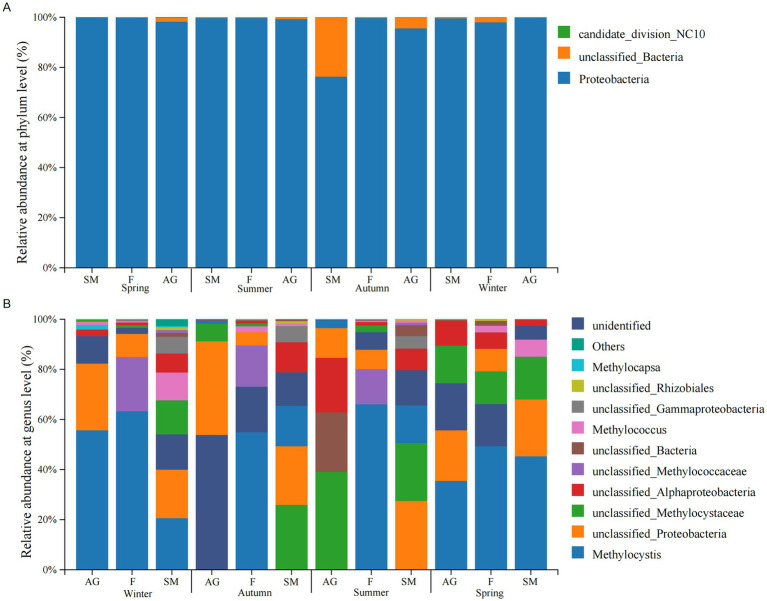
Community structure composition at phylum level **(A)** and genus level **(B)** of methanotrophs in wetlands of Mitika in different seasons.

The dominant bacterial phyla detected in soil samples from the typical wetland ecosystem of Mitika across different seasons were Proteobacteria (aerobic methanotrophs) (76.33–99.90%), candidate_division_NC10 (0.03%), and unclassified bacteria (0.09–22.3%) (Figure 4A). Proteobacteria was present in all seasonal soil samples from the typical Mitika wetland, with the lowest relative abundance observed in autumn marsh meadows. Candidate_division_NC10 appeared only in small quantities in autumn and winter marsh meadows. Candidate_division_NC10 belongs to anaerobic methanotrophs and is the primary microorganism in the nitrite-type anaerobic methane oxidation process.

#### 3.3.2 Methanotrophs community composition at the genus level

To further investigate the composition of the phylum Proteobacteria in the study area soils, we conducted additional analyzes of methanotrophs at the genus level. The genus-level community structure was dominated by *Methylocystis*, *Methylococcus*, *Methylocapsa*, and others (Figure 4B). *Methylocystis* (1.3–63%), unclassified_Proteobacteria, unclassified_Methylocystaceac, unclassified_Alphaproteobacteria were distributed across typical wetland ecosystems in different seasons. *Methylocystis* exhibited higher relative abundance in spring and winter, and its abundance was lower in autumn alpine meadows and summer marshy meadows. Numerous methanotrophs remained undetected in autumn alpine meadows, including unclassified_Methylococcaceae, *Methylococcus*, *Methylocapsa*, and unclassified_Rhizobiales. *Methylococcus* was absent from alpine meadows in spring and autumn; *Methylocapsa* was not detected in typical wetland ecosystems during spring or in swamp meadow during summer and autumn, exhibiting low relative abundance. Based on genus-level classification of methanotrophs, the Proteobacteria in typical wetland ecosystems of the Mitika system belong to Type II, specifically *α*-Proteobacteria, primarily utilizing the serine cycle for formaldehyde assimilation within the inner membrane and periplasmic space (Table 2).

**Table 2:** Classification of methanotrophs.

Type		Phylum	Genus	Function
Aerobic methane-oxidizing bacteria	I, X	γ-Proteobacteria	*Methylomonas*, *Methylobacter*, *Methylosarcina, Methylomicrobium, Methylohalobius, Methylosphaera, Methylosoma, Methylothermus, Methylocaldum, Methylococcus*	Formaldehyde assimilation via the ribulose monophosphate pathway on the endoplasmic reticulum membrane
	II	α-Proteobacteri	Methylosinus, Methylocystis, Methylocella, Methylocapsa, Methyloferula	Formaldehyde assimilation via the serine cycle in the endoplasmic reticulum and periplasmic space
	Others	Verrucomicrobia	Met hylokorus infer norum[8], Aci dimethylosilex fumarolicum[9], Met hyloacida kamchatkensis	Extremophile Acetobacter
Anaerobic methane-oxidizing bacteria	Archaea	Anaerobic methane-oxidizing archaea	ANME-1, ANME-3, ANME-2a, ANME-2b, ANME-2c	Co-perform sulfate-dependent anaerobic methane oxidation with sulfate-reducing bacteria
			ANME-2d	Co-perform sulfate-dependent anaerobic methane oxidation with sulfate-reducing bacteria
	Bacteria	NC10	Candidatus Methylomirabilis oxyfera	Primary Microorganisms in the Nitrite-Type Anaerobic Methane Oxidation Process

### 3.4 Assembly process of methanotrophs communities across seasons

**Figure 5:**
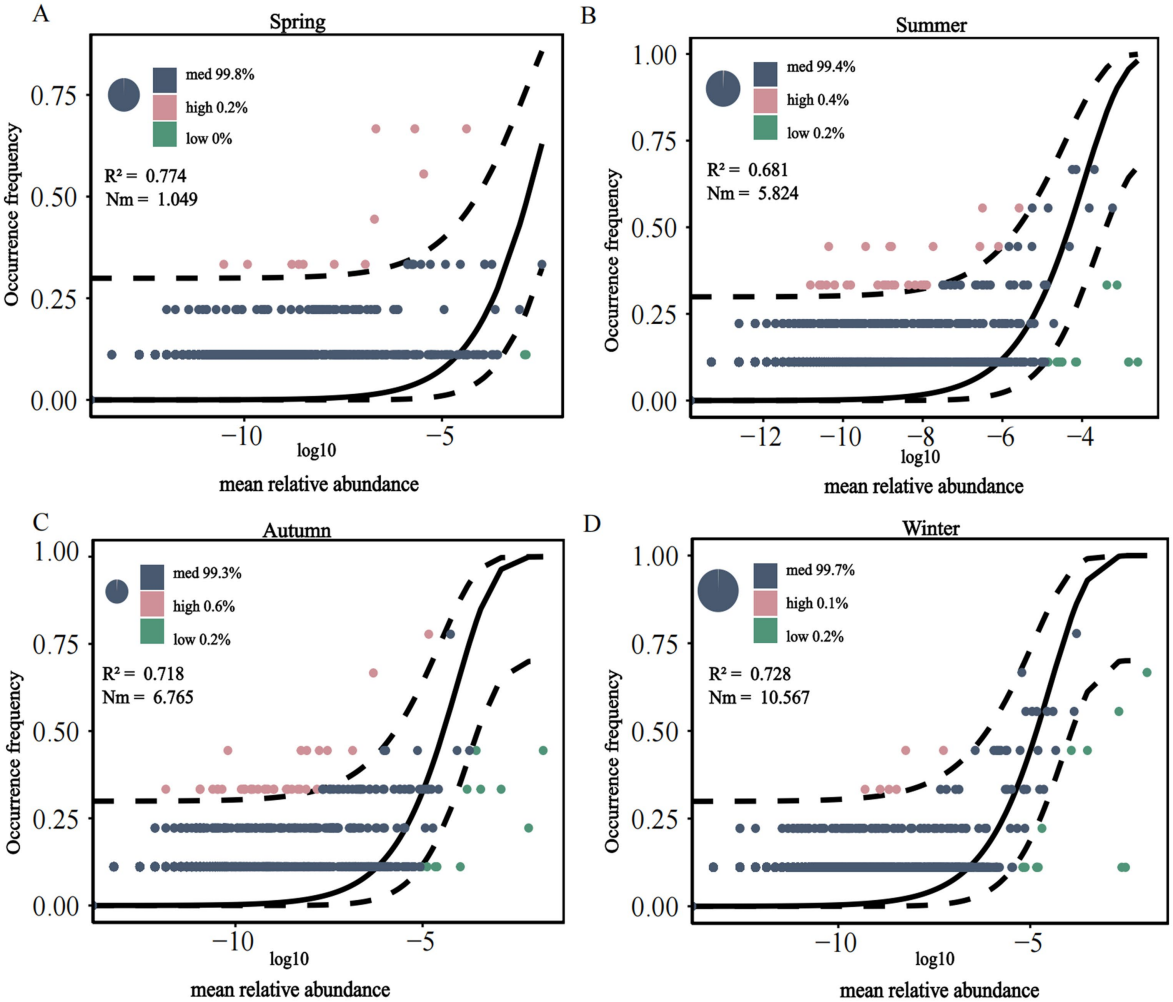
Neutral model of soil methanotrophs community in alpine wetland of Mitika in different seasons (**A**–**D**).

The assembly processes of methanotrophs communities across seasons were analyzed using the NCM to distinguish between random and deterministic processes, with R^2^ representing the model goodness-of-fit (Figure 5). The overall fit of the neutral model for the soil methanotrophs community in the Mitika alpine wetland showed the following seasonal order: spring (0.774) > winter (0.728) > autumn (0.718) > summer (0.681). This indicates that the winter soil fungal community more closely conformed to the neutral model, being influenced by both random and deterministic processes, with random processes playing a dominant role in shaping community structure. The NCM parameter reflects the scale of individual migration between locations during population succession, with its value positively correlated to migration rate. The trend indicating more uniform distribution of soil methanotrophs communities in winter, which facilitates species dispersal. This indicates that the winter soil methanotroph community more closely conformed to the neutral model, being influenced by both random and deterministic processes, with random processes playing a dominant role in shaping community structure. Overall, most methanotrophs fell within the medium-abundance range, whereas species in the high- and low-abundance ranges were relatively scarce.

### 3.5 Effects of soil environmental factors on methanotrophs communities

**Figure 6:**
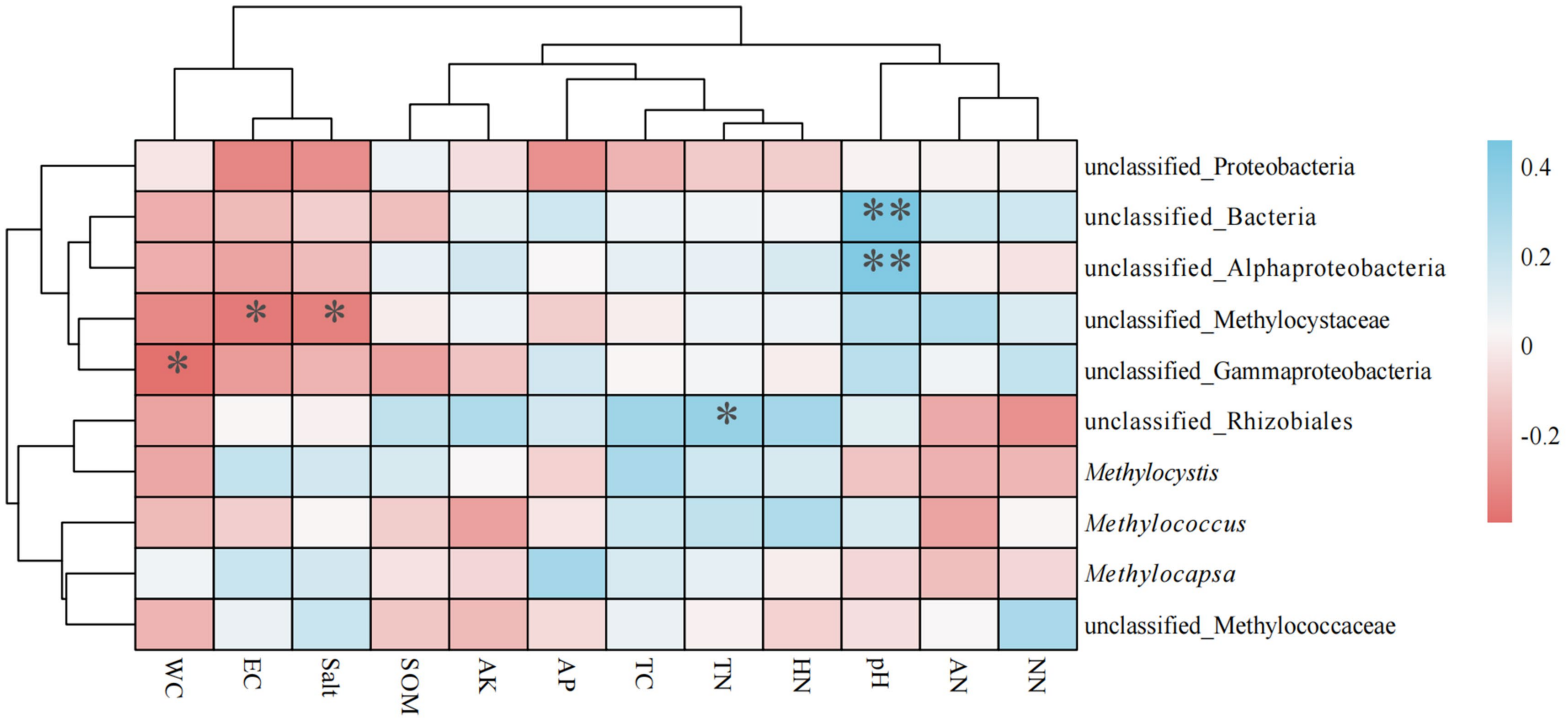
Heat map of correlation between methanotrophs and environmental factors.

Spearman’s rank correlation coefficients between soil physicochemical factors and genus-level methanotrophs revealed significant differences in the influence of environmental factors on microbial communities (Figure 6). Three genera exhibited significant negative correlations with environmental factors, whereas two genera showed significant positive correlations. Specifically: unclassified_Bacteria and unclassified_Alphaproteobacteria showed extremely significant positive correlations with pH (*p* < 0.01); unclassified_Rhizobiales exhibited a significant positive correlation with TN; unclassified_Methylococcaceae demonstrated significant negative correlations with EC and Salt; and unclassified_Gammaproteobacteria showed a significant negative correlation with WC.

**Figure 7:**
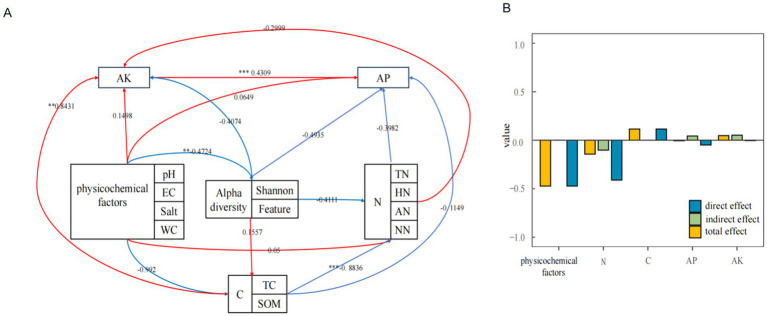
Structural equation modeling of methanotrophs and environmental factors **(A)** and effect calculations **(B)**.

To further investigate the effects of environmental factors on community structure, environmental factors were categorized into basic soil physicochemical factors, carbon cycle-related environmental factors, nitrogen cycle-related environmental factors, available phosphorus, and available potassium. Structural equation modeling was employed to analyze the influence of these classified environmental factors on alpha diversity (Figure 7A). Furthermore, direct, indirect, and total effect values between alpha diversity and environmental factors were calculated (Figure 7B). Basic soil physicochemical factors were found to be significantly negatively correlated with alpha diversity indices (*p* < 0.001). Alpha diversity indices correlated with all other environmental factors, exhibiting direct effects only with basic physicochemical and carbon cycle-related factors, and indirect effects only with readily available phosphorus and potassium.

## 4 Discussion

Wetlands represent a significant source of atmospheric methane, a major greenhouse gas second only to CO₂ (Helfter et al., 2022). Investigating methanotrophs in wetlands is crucial for understanding and predicting global methane emission dynamics, assessing the role of wetlands in climate change, and developing effective greenhouse gas reduction strategies. Our analysis identified two dominant methanotroph phyla in the Mitika alpine wetland: Proteobacteria and candidate division NC10. This finding aligns with Deng et al. regarding Proteobacteria’s dominance but extends it by identifying a detectable, albeit low-abundance (Deng et al., 2015), NC10 community. The divergence from studies where NC10 was not prominent may stem from this site’s unique high-altitude conditions (e.g., cooler temperatures, heightened UV radiation), which could favor a more diverse consortium, including niche-adapted NC10 methanotrophs. Methodological differences, such as primer specificity, could also influence NC10 detection. Thus, the presence of NC10 here does not contradict but rather complements the established paradigm, underscoring that ecosystem-specific factors and technical approaches are crucial for revealing the full spectrum of ecologically significant methanotrophs beyond the core Proteobacteria. To more precisely identify the type of Proteobacteria in the study soils, we further analyzed the samples at the genus level. By comparing our results with the findings of Cai et al. (2016), we found that the methanotrophs detected in this study belong to Type II methanotrophs, which primarily belong to *α*-Proteobacteria. Type II methanotrophs primarily utilize the serine pathway within the inner membrane and periplasmic space to assimilate formaldehyde. This serine pathway is a crucial metabolic pathway enabling microorganisms to convert formaldehyde produced during methane oxidation into organic compounds, which are then utilized for cellular growth and reproduction. This discovery holds significant implications for understanding the diversity and function of methanotrophs within wetland ecosystems. This study reveals candidate division NC10 as a functionally significant methanotroph alongside Proteobacteria in the Mitika alpine wetland, indicating broader diversity and adaptive methane oxidation under environmental stresses. While consistent with Zhang et al. regarding higher diversity in swamp meadows (Zhang et al., 2019), our observed greater overall diversity likely reflects methodological advances and regional specificity. Limited concurrent environmental data represents a constraint. Future work integrating metagenomics could verify *in situ* activity. These findings improve predictions of methane emissions from vulnerable alpine ecosystems, supporting targeted climate mitigation strategies.

In general, methanotrophs primarily exhibit negative correlations with environmental factors, such as WC, EC, and salt, and positive correlations with pH, TN, and HN. The correlations observed in the present study correspond with this general pattern. Methanotrophs exhibit methane oxidation activity in both acidic and alkaline habitats, with aerobic methanotrophs being prevalent in acidic environments (Yao et al., 2023). In the present study, aerobic methanotrophs (α-Proteobacteria) and anaerobic methanotrophs (NC10) were detected. Currently, few published studies have examined the effects of pH on anaerobic methanotrophs. In the 1980s, several genera of aerobic methanotrophs-*Methylosinus*, *Methylocella*, *Methylocystis*, *Methylocapsa*, *Methylobacter*, *Methylomonas*, and *Methylovulum*-were isolated from acidic peatlands in the Northern Hemisphere (Whittenbury et al., 1970). Our results showed non-significant positive/negative correlations between SOM and methanotrophs, differing from the strong positive correlation reported by Lee et al. in coniferous forests (Lee et al., 2023). This discrepancy is likely due to ecosystem differences and variable sampling times.

Notably, this study identified Methylobacter and NC10 phylum as the dominant methanotrophic functional groups in the Maidiqia wetland, a composition pattern significantly different from low-altitude wetlands. In Qinghai-Tibetan Plateau lake wetlands, Methylobacter maintains methane oxidation activity under anoxic conditions through its high-affinity cytochromes and fermentation metabolic flexibility (Schorn et al., 2024). In contrast, at lower-elevation freshwater wetlands (e.g., Old Woman Creek), Methylobacter also dominates methane oxidation but exhibits greater metabolic dependence on aerobic conditions (Smith et al., 2018).

This functional group differentiation along the altitudinal gradient may stem from variations in temperature, oxygen partial pressure, and nitrogen source availability: high-altitude wetlands exhibit year-round low temperatures, oxygen deficiency, and nitrogen limitation, compelling the mixed acid fermentation pathway of Methylobacter to evolve survival strategies increasingly reliant on denitrification and fermentation metabolism (Yun et al., 2021; Hao et al., 2013). Furthermore, the coexistence of Methylobacter and NC10 in the Zoige wetland suggests that high-altitude environments may enhance methane filtration efficiency by promoting functional group complementarity through niche differentiation—such as aerobic oxidation in surface layers and anaerobic oxidation in deeper layers (Xie et al., 2020).

The community structure characteristics of high-altitude wetland methanotrophs revealed in this study are highly consistent with the findings of the aforementioned multiple studies. The functional complementarity arising from niche differentiation between Methylobacter and NC10 bacteria supports our conclusion that high-altitude wetland methanotrophs employ unique adaptive strategies. This not only validates the credibility of our findings but also highlights the distinctive ecological role and carbon cycling function of these methanotroph communities. These insights provide crucial theoretical foundations for understanding microbial regulation mechanisms governing greenhouse gas emissions from alpine wetlands under global change.

## 5 Conclusion

In conclusion, this study demonstrates that the community structure and diversity of soil methanotrophs in the Mitika alpine wetland are primarily shaped by ecosystem type, with a secondary yet significant influence from seasonal variation. Swamp meadows host the most diverse communities, and stochastic processes dominate the community assembly, particularly in winter. The overall composition is strongly dominated by Type II methanotrophs (*α*-Proteobacteria). Critically, our findings identify pH, Total Nitrogen (TN), Electrical Conductivity (EC), Salt, and Water Content (WC) as the key environmental drivers governing methanotroph distribution and diversity in this high-altitude ecosystem. Structural equation modeling further confirms the significant positive influence of these fundamental soil physicochemical factors on community alpha diversity. These insights advance our understanding of microbial mediation in carbon cycling processes in vulnerable alpine wetlands under changing climatic conditions.

## Data Availability

The datasets presented in this study can be found in online repositories. The names of the repository/repositories and accession number(s) can be found in the article/supplementary material.
